# Supplementary material for: Association between radiographic severity with health-related quality of life in elderly women with knee osteoarthritis: A cross-sectional study
Source: PLoS One. 2025 May 8;20(5):e0319482. doi: 10.1371/journal.pone.0319482 (PMC12061131; doi:10.1371/journal.pone.0319482)
Supplement: Attachment — Submitted filename: Response to Reviewers.docx [file pone.0319482.s005.docx]

**A rebuttal letters**

**Dear Editor and Reviewers,**

We would like to express our sincere gratitude for the time and effort editor and the reviewers have spent on evaluating our manuscript titled " Association between radiographic severity with health-related quality of life in elderly women with knee osteoarthritis: A cross-sectional study". We truly appreciate the constructive feedback and thoughtful suggestions provided, which have significantly improved the quality of our work.

In response to the editor and reviewers' comments, we have carefully revised the manuscript to address the concerns raised. Below is a summary of the major revisions we made:

**Journal requirements:**

**1. Please ensure that your manuscript meets PLOS ONE's style requirements, including those for file naming.**

Thank you for the remainder from the editor. We have adjusted the manuscript according to the PLOS ONE style templates to ensure that all file naming complies with the journal's formatting requirements. The necessary revisions have been completed, and the updated files are attached.

**2. We note that the grant information you provided in the ‘Funding Information’ and ‘Financial Disclosure’ sections do not match.**

Thank you for your feedback. We have verified the funding information and updated the "Funding Information" section.

**3. We note that your Data Availability Statement is currently as follows: [All relevant data are within the manuscript and its Supporting Information files.]**

According to PLOS ONE requirements, we have uploaded the minimal data set, which includes the raw data used to calculate means, standard deviations, and other statistical measures, as well as the values used to build graphs and the points extracted from images for analysis. We have deposited the data in the Dryad repository, and the relevant DOI is: <http://datadryad.org/stash/share/zdrxjMWFkIjpRK4MB8XrQ9RzLDDO2gd6h0Uwh4pW5DA>. The DOI link is: <https://doi.org/10.5061/dryad.sj3tx96ff>. Please feel free to contact us if you have any questions.

**4. Your ethics statement should only appear in the Methods section of your manuscript. If your ethics statement is written in any section besides the Methods, please move it to the Methods section and delete it from any other section.**

Thank you for the reminder. We have moved the ethics statement from other sections of the manuscript to the "Methods" section and ensured that the ethics statement appears only in the "Methods" section. Please refer to the resubmitted manuscript.

**5. Please include a separate caption for each figure in your manuscript.**

Thank you for your feedback. We have provided a separate caption for each figure to ensure that all figure descriptions are clear and accurate. The revised manuscript has been updated, please refer to it.

**6. Please include your tables as part of your main manuscript and remove the individual files. Please note that supplementary tables (should remain/ be uploaded) as separate "supporting information" files**

Thank you for your suggestion. We have uploaded all tables as part of the main manuscript and removed the separate table files.

**7. Please review your reference list to ensure that it is complete and correct.**

Thank you for your reminder. We have rechecked the reference list to ensure its accuracy and completeness.

**Reviewer #1:**

**1) In the sentence “However, some studies have reported that the association between the radiographic severity of knee OA and clinical outcomes remains controversial [18].”, you have used plural expression by writing “some studies” although you have only one reference. Please review this again.**

Thank you for your feedback. It was an oversight on our part, and we have now added additional relevant studies in this section to support the argument.

**2) Improve your manuscript English. Use subjects more specifically in sentences instead of words like they, their, it. If necessary, you can utilize the translation service.**

Thank you for your suggestion. We have carefully reviewed the manuscript and have avoided using ambiguous pronouns, ensuring that the subject of each sentence is clear and specific to improve the accuracy and readability of the language.

**3) Indicate which statistical analysis you utilized to determine whether there is a normal distribution.**

Thank you for your reminder. In the methods section, we have added that the Shapiro-Wilk test was used to assess the normality of the data, the specific method is described in the text.

**4) In the limitations section, state that the results of the present study cannot be generalized to the male gender and to individuals younger than 50 years of age.**

Thank you for your valuable suggestion. We have clearly stated in the limitations section that the study results are only applicable to specific age groups and genders, and therefore cannot be generalized to the male population or individuals under the age of 50. Future studies should include more diverse populations to enhance the applicability of the findings.

**5) In your findings, the body mass index is significantly higher in the group with KL 3-4 than in the other group. This may have negatively affected quality of life and other outcome measures, regardless of the stage of CL. How did you control for the possible negative effect of body mass index in the comparison between groups?**

Thank you for your question. We used Analysis of Covariance (ANCOVA) in the data analysis to control for the potential effect of BMI in the comparisons between groups. We have provided a detailed explanation of this in the methods section to ensure that the impact of BMI is appropriately controlled.

**Reviewer #2:**

**1-The authors should provide a calculation-based sample size analysis.**

Thank you for your suggestion. We have added detailed information about the sample size calculation in the methods section. Based on the expected effect size and statistical power analysis, we determined the required sample size to ensure sufficient statistical power for the study results.

**2-Clinical implications section should be added to the discussions section to improve the practical implications of the results.**

Thank you for your suggestion. We have added a section on clinical implications in the discussion, exploring the potential impact of the study’s findings on clinical practice and how they can guide future research.
